# Thymosin Beta 4 May Translocate from the Cytoplasm in to the Nucleus in HepG2 Cells following Serum Starvation. An Ultrastructural Study

**DOI:** 10.1371/journal.pone.0119642

**Published:** 2015-04-02

**Authors:** Marco Piludu, Monica Piras, Giuseppina Pichiri, Pierpaolo Coni, Germano Orrù, Tiziana Cabras, Irene Messana, Gavino Faa, Massimo Castagnola

**Affiliations:** 1 Department of Biomedical Sciences, University of Cagliari, Cagliari, Italy; 2 Divisione di Anatomia Patologica, Dipartimento di Citomorfologia, University of Cagliari, Cagliari, Italy; 3 Dipartimento di Scienze della Vita e dell’Ambiente, Universitadi Cagliari, Cagliari, Italy; 4 OBL, Department of Surgical Sciences, University of Cagliari, Cagliari, Italy; 5 Istituto di Biochimica e di Biochimica Clinica, Universita`Cattolica, Roma, Italy; 6 Istituto per la Chimica del Riconoscimento Molecolare, CNR, IstitutoScientifico, Internazionale (ISI) Paolo VI, Roma, Italy; Federal University of Rio de Janeiro, BRAZIL

## Abstract

Due to its actin-sequestering properties, thymosin beta-4 (Tβ4) is considered to play a significant role in the cellular metabolism. Several physiological properties of Tβ4 have been reported;, however, many questions concerning its cellular function remain to be ascertained. To better understand the role of this small peptide we have analyzed by means of transmission immunoelectron microscopy techniques the ultrastructural localization of Tβ4 in HepG2 cells. Samples of HepG2 cells were fixed in a mixture of 3% formaldehyde and 0.1% glutaraldehyde in 0.1 M cacodylate buffer and processed for standard electron microscopic techniques. The samples were dehydrated in a cold graded methanol series and embedded in LR gold resin. Ultrathin sections were labeled with rabbit antibodies to Tβ4, followed by gold-labeled goat anti-rabbit, stained with uranyl acetate and bismuth subnitrate, observed and photographed in a JEOL 100S transmission electron microscope. High-resolution electron microscopy showed that Tβ4 was mainly restricted to the cytoplasm of HepG2 growing in complete medium. A strong Tβ4 reactivity was detected in the perinuclear region of the cytoplasmic compartment where gold particles appeared strictly associated to the nuclear membrane. In the nucleus specific Tβ4 labeling was observed in the nucleolus. The above electron microscopic results confirm and extend previous observations at light microscopic level, highlighting the subcellular distribution of Tβ4 in both cytoplasmic and nuclear compartments of HepG2 cells. The meaning of Tβ4 presence in the nucleolus is not on the best of our knowledge clarified yet. It could account for the interaction of Tβ4 with nucleolar actin and according with this hypothesis, Tβ4 could contribute together with the other nucleolar acting binding proteins to modulate the transcription activity of the RNA polymerases.

## Introduction

The Beta-thymosins family comprises 16 known members with an highly conserved amino-acid sequence in species ranging from mammals to echinoderms. Among these, thymosin beta 4 (Tβ4) is the most abundant member in human cells and tissues, representing approximately 70–80% of the total thymosin content [1–3]

Several physiological properties and cellular functions of Tβ4 have been described. Tβ4 is the major actin-sequestering molecule in all eukaryotic cells and a potent regulator of actin polymerization in mammals [4]. This small peptide may also have activities independent from the G-actin-binding properties: its localization and its dynamic, unstructured and flexible conformation seem to be determinant [5].

To better understand the role of this small peptide, several studies have analyzed in detail its intracellular localization. Tβ4 subcellular localization was described to be either cytoplasmic or nuclear and cytoplasmic, according to different cells and tissues. In resting macrophages, immunoreactivity for Tβ4 was found to be restricted to the cytoplasm, in the absence of any nuclear immunostaining [6]. Cytoplasmic and nuclear positivity was found with labelled Tβ4 injected into *Xenopus laevis* oocytes [7]. Variable Tβ4 cytoplasmic immunoreactivity was found constantly associated with nuclear staining in the human mammary carcinoma MCF-7 cell line [8]. Polyamine depletion in migrating IEC-6 cells and ischemia in the rat brainhave been shown to induce a translocation of Tß4 into the nucleus[8]. The nuclear Tβ4 localization seems to be of particular interest regarding its multiple functions and remains to be ascertained, at the best of our knowledge, the significance of this phenomenon is not clear. Experiments with microinjection of two fluorescently labeled Tβ4 fragments into HeLa cells supported the hypothesis of the existence of a specific active transport mechanism regulating translocation of this peptide into the cell nucleus[9]. On the contrary, another study using different Tβ4 variants underline a possible passive but regulated diffusion mechanism, suggesting that Tβ4 translocation could be regulated by the change of the pore permeability [10].

Another example of Tβ4 intracellular trafficking was recently described in HepG2 cells under starvation, suggesting that Tβ4 might be able to translocate from different cytoplasmic domains into the nucleus and back, based on different stress conditions within the cell [11].

Since no data regarding Tβ4 ultrastructural localization are available, in this study we performed electron microscopy immunostaining in HepG2 cells in normal condition and under 48h of starvation conditions in order to better clarify the cytoplasm-nuclear translocation previously described. Tβ4 mRNA expression during starvation was also analyzed in different experimental conditions.

## Materials and Methods

### Cell culture

Commercial human cell line HepG2 (ICLC HTL95005) were obtained from the Istituto Nazionale per la Ricerca sul Cancro c/o CBA (ICLC, Genova). The culture medium used for this purpose was a mixture of MEM (EBSS), 10% fetal bovine serum (FBS), 100 units/ml penicillin, 100 mg/ml streptomycin, 2 mM L-Glutamine, 1% non-essential amino acids. To perform different experimental conditions, confluent cells were isolated using trypsin/EDTA and, for the experimental procedure, samples of 2–3 x 10^4^ cells/cm2 HepG2 cells were plated on different glass coverslips at 37°C, 5% CO_2_. After 24 h of growth with complete medium, cells were cultured with complete culture medium or with medium without FBS for 48 h. All samples were washed with PBS and HepG2cells in normal serum and after starvation were collected after trypsin detachment.Experiments were repeated 3 times

### Ultrastructural analysis

In this study a post-embedding immunogold staining (IGS) method was used. Samples of HepG2 cell cultures were fixed in a mixture of 3% formaldehyde and 0.1% glutaraldehyde in 0.1 M cacodylate buffer and processed by standard methods for embedding in LR Gold resin.

Ultrathin sections (90 nm thick) collected on formvar-coated nickel grids were floated section-side down on phosphate-buffered saline (PBS) for 5 min, then transferred to small drops (30 μl) of PBS containing 1% bovine serum albumin (BSA) and 5% normal goat serum (NGS) for 20 min at room temperature to block non-specific binding. The sections were incubated in a humidified chamber overnight at 4°C with a rabbit polyclonal antibody reactive for Tβ4 (Bachem-Peninsula Lab, San Carlos, CA, USA).

Sections incubated with medium devoid of primary antibody or containing non-immune serum were used as controls. After flushing with PBS, the grids were incubated for 60 min at room temperature with the secondary antibody, gold-labeled goat anti-rabbit IgG (Auroprobe EM, Amersham International PLC, Little Chalfont, UK), diluted 1:50 in 1% BSA–PBS. The grids were washed with PBS and distilled water, observed and photographed in a transmission electron microscope (JEOL 100S model, Jeol, Tokyo, Japan) operating at 80 kV.

### Real Time RT-PCR

As previously described [12], cells were immediately frozen in dry ice, and kept at -70°C until lysis for RNA extraction. Total RNA was extracted using the Qiagen RNeasy Mini Kit (Qiagen) according to manufacturer’s instructions. The human β-actin was used as referencehousekeeping gene [13].

The following primers (b-actF = 5'-GCATGGGTCAGAAGG-3', b-actR. = 5'-AGGCGTACAGGGATAG-3', tb4F = 5'-GGCCACTGCGCAGACCAGACT3' tb4R. = 5'CTTGATCCAACCTCTTTGCATCTTACAA-3') were designed using the sequences of the Tβ4 RNA (GenBank accession no. NM_001101) and the human beta- actin mRNA (GenBank accession no. NM_001101).

Real-time reverse-transcriptase PCR analysis was performed in a Light Cycler apparatus (Roche) with a LightCycler-RNA using the SYBR Green I amplification kit (Roche Diagnostics) according to the manufacturer’s instructions. The 20 ml final volume contained: 3 mM MgCl_2_, 0.25 mM of each primer, 2 ml of RNA extract. Cycling was performed using the following amplification conditions: an initial reverse transcription at 55°C for 10 min, denaturation at 95°C for 30 sec followed by 35 cycles at 95°C for 10 sec, 53°C for 10 sec and 72°C for 8 sec with subsequent melting analysis: heating to 95°C for 20s, cooling to 45°C for 10 sec and reheating to 95°C at a rate of 0.2°C per second.

Fluorescence was detected at the end of the 81°C segment in PCR step (single mode) and at 45°C segment in the melting step (continuous mode) in the F1 channel. The relative gene expression was analyzed by using the 2-DDCT method [14]. For each analysis, three distinct biological replicas were done, and quantitative data were expressed as mean. Values of fold change in Tβ4 gene expression relative to the beta-actin has been represented as mean+standard error.

## Results

### HepG2 ultrastructural localization of Tβ4 in normal conditions and under starvation.

Since our previous experiments were performed by means of light microscopy, we used transmission electron microscopy in order to add further details on Tβ4 localization in HepG2 cells at different cell conditions. The higher resolving power of the electron microscopic technique highlighted the ultrastructure of HepG2 cells that were characterized by the presence of a well-developed endoplasmic reticulum and Golgi apparatus in the cytoplasm and by prominent nucleoli in the nuclear compartment. The nuclear envelope structure was conformed to previous descriptions [15], being characterized by the presence of two concentric lipid bilayers, the inner and the outer membranes separated by the intramembranous space and by evident nuclear pores.

**Fig 1:**
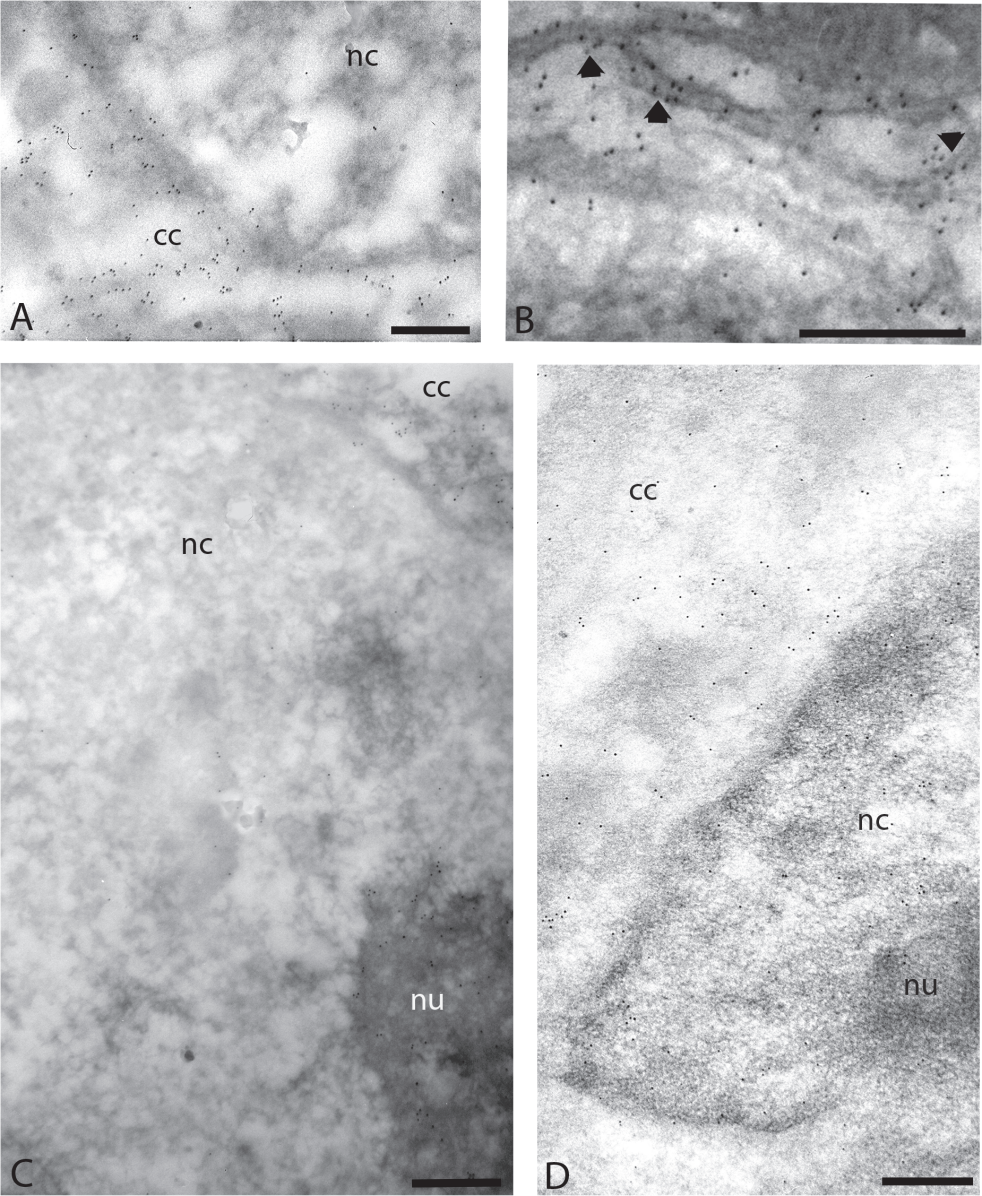
(A-C) Electron micrographs of HepG2 cells growing in complete medium. Specific Tβ4 reactivity is detected in the cytoplasmic compartment (cc), where gold particles are observed strictly associated to the endoplasmic reticulum (arrows). In the nuclearcompartment (nc) the nucleoplasm is devoid of labeling whereas the nucleolus (nu) shows evident labeling. **(D) Portion of HepG2 cell growing for 48h in the absence of fetal bovine serum.** Specific Tβ4 immunostaining is observed in both cytoplasm (cc) and nucleoplasm (nc). On the contrary, few gold particles decorate the nucleolus (nu). Bars = 0,5 μm

Immunoelectron microscopy showed significant differences regarding Tβ4 localization in the cellular compartments of HepG2 cells under different cell conditions. HepG2 cells growing in the complete medium were characterized by evident Tβ4 expression in the cytoplasm (Fig. 1A and 1C). The perinuclear region of the cytoplasmic compartment was characterized by a strong Tβ4 staining, where gold particles were detected to be strictly associated to the endoplasmic reticulum (Fig. 1B). In the nuclear compartment, nucleoplasm appeared unreactive or weakly labeled (Fig. 1A and 1C), whereas the nucleolus resulted frequently labeled for Tβ4 (Fig. 1C).

In HepG2 cell cultures growing for 48h in the absence of fetal bovine serum, evident Tβ4 immunostaining was observed in both cytoplasm and nucleus (Fig. 1D). In particular, in the nuclear compartment Tβ4 was uniformly distributed in the nucleoplasm and only few gold particles decorated occasionally the nucleolus (Fig. 1D).

### Tβ4 RNA expression in HepG2 cells under starvation.

We have assumed that during mRNA expression analysis after 24h in normal condition, the ratio Tβ4/β actin was equal to 1 (calibrator sample) [14].

**Fig 2:**
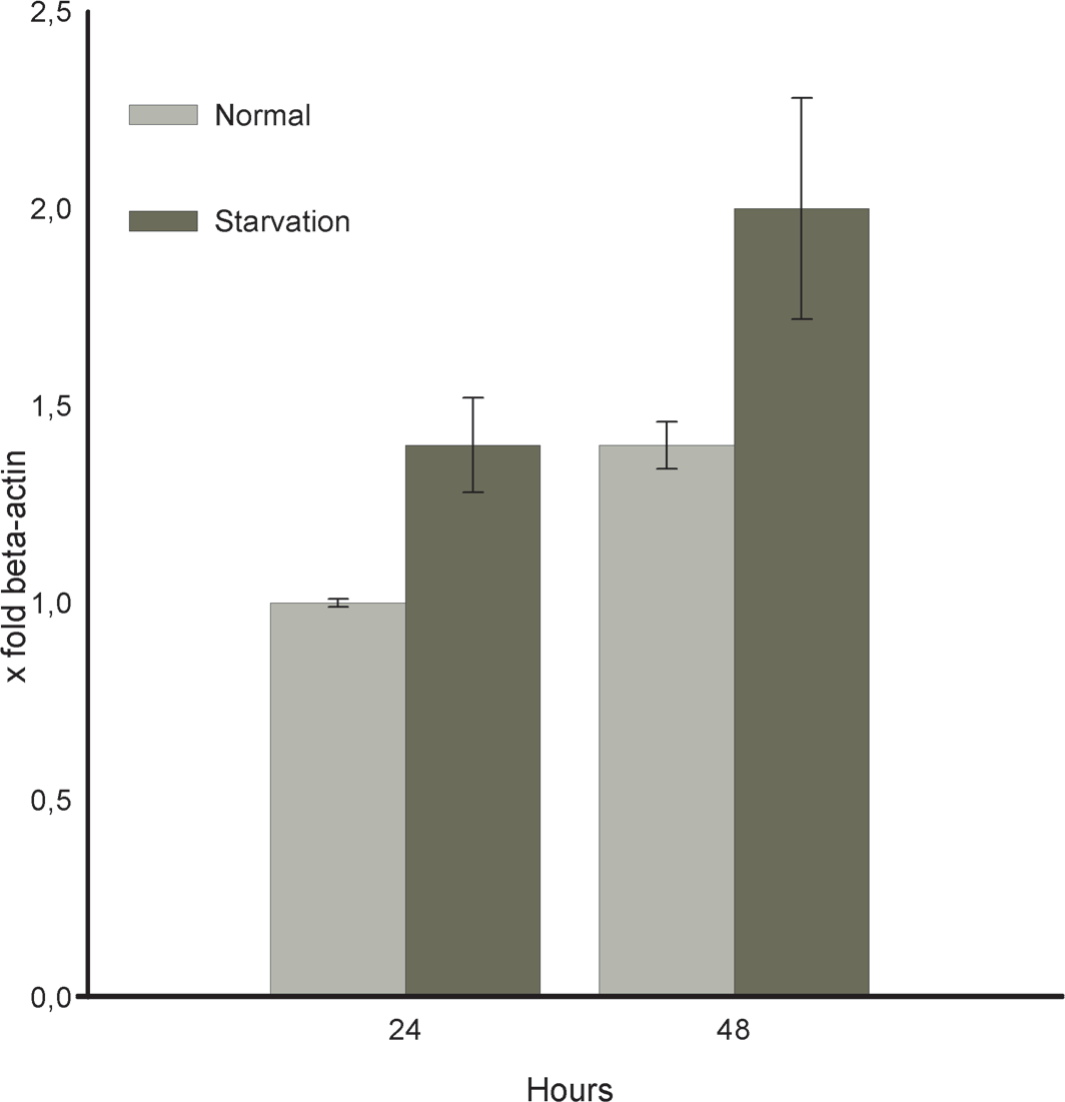
Tβ4 mRNA expression detected by RT-PCR reaction in HepG2 cells growing in normal serum and in starvation conditions. The ratio value Tβ4/β actin detected at 24h in normal condition was assumed as equal to 1 (calibrator sample). In these conditions the Tβ4 expression patterns suggest a moderate Tβ4 mRNA increase expression rate from 24 to 48h in normal conditions and, a more high Tβ4 mRNA expression values, during starvation.(2 folds in difference at 48h). The vertical bars, reppresent the range of standard error (+-SE) of the mean.

In these conditions the Tβ4 expression patterns suggest a moderate increase of the Tβ4 expression rate from 24 to 48h in normal conditions and, a more high Tβ4 expression values, during starvation.(2 folds in difference at 48h). This observation confirm that Tβ4 may be involved in stress processes, not only changing its intracellular localization, but also affecting its gene expression (Fig. 2).

## Discussion

In this study we analyzed at high-resolution level the distribution of Tβ4 in the cellular compartments of HepG2 cells in different cell conditions. The peculiar Tβ4 immunoreactivity pattern here observed raises interesting questions concerning the physiological properties of Tβ4. Tβ4 was mainly localized in the cytoplasm of HepG2 cells growing in complete medium, whereas in the nuclear compartment Tβ4 reactivity was restricted inside the nucleolus. In starving HepG2 cells, Tβ4 changes its intracellular distribution, being detected both in cytoplasm and nucleoplasm.

The electron microscopic results confirmed and extended previous observations at light microscopic level [11]. Our ultrastructural data point out that Tβ4 nuclear translocation may occur in both normal and starving conditions, confirming and highlighting the important role of the nuclear membrane in the regulation of Tβ4 exchange process from cytoplasm to nucleus. It’s well known that the nuclear envelope plays a fundamental role in the regulation of protein traffic from cytoplasm to the nuclear compartment and back. Nuclear membrane envelop is mainly described as tripartite structure being characterized by the presence of two concentric lipid bilayers, the inner and the outer membranes being separated by the intramembranous space [16]. High-resolution electron microscopy showed how most Tβ4 was restricted to the cytoplasm of HepG2 cells growing in complete medium. Strong Tβ4 reactivity was also detected at the perinuclear region strictly associated to the nuclear envelope. Actually, it is not clear the meaning of such localization but it may underliea specific interaction of Tβ4 with the nuclear pores during its transfer process into the nuclear compartment. Nuclear pores that pierce the nuclear envelope regulate the protein exchange between the cytoplasm and nucleus [17] and they are believed to be involved in the translocation of Tβ4 from the cytoplasm compartment to the nucleus [18]. Previous observations at light microscopy level under starvation have provided additional findings regarding the Tβ4 nuclear translocation in HepG2 cells, highlighting the presence of Tβ4 in the nucleus envelope as punctuated reactivity that were suggested to be related to the nuclear pores [11]. Despite the fact that the nuclear envelope is usually permeable to small peptides andmetabolites, molecules with a greater mass need to be actively shuttled [19]. Several proteins are imported in the nucleus by specific transport molecules that regulate cytoplasmic-nuclear exchange [15,20]. Recently, it has been suggested that Tβ4 could be shuttled into the nucleus by an active transport mechanism [21] through an unidentified soluble factor [22].

The presence of Tβ4 in the nucleus suggest that it might play a significant role in cellular metabolism and could account for its involvement in the regulation of gene transcription. Due to the higher resolving power of the electron microscopic technique used in this study, we were able to demonstrate the differential expression of Tβ4 in the nuclei of HepG2 cells in different cell conditions. Tβ4 appeared restricted to the nucleolus of the cells growing in complete medium, whereas in HepG2 cells under starvation it was mainly distributed in the nucleoplasm and only few gold particles decorated occasionally the nucleolus. These results suggest the existence of a specific Tβ4 nuclear trafficking from nucleoplasm to nucleolus and add additional information concerning its possible role in cellular metabolism. Previous studies have investigated the possible molecular mechanisms leading to the nucleolar translocation of non-ribosomal proteins, trying to understandifthey were routed to the nucleolus through an active transport or a simple diffusion. Recently, it has been reported the interaction of the actin filament capping proteins with specific molecules that are required to target to the nucleolus. The nucleolar actin capping protein CapG has been shown to translocate in the nucleus by a specific transport receptor [15] and to be shuttled by an ATP-dependent translocation pathway to the nucleolus [23]. Once transported in the nucleus, Tβ4 could be recruited to the nucleolus through specific interactions with other molecules that shuttle between the nucleoplasm and nucleolus depending on an ATP-dependent translocation pathway. The changed environmental conditions could alter this process, preventing the localization of Tβ4 in the nucleolus.

**Fig 3:**
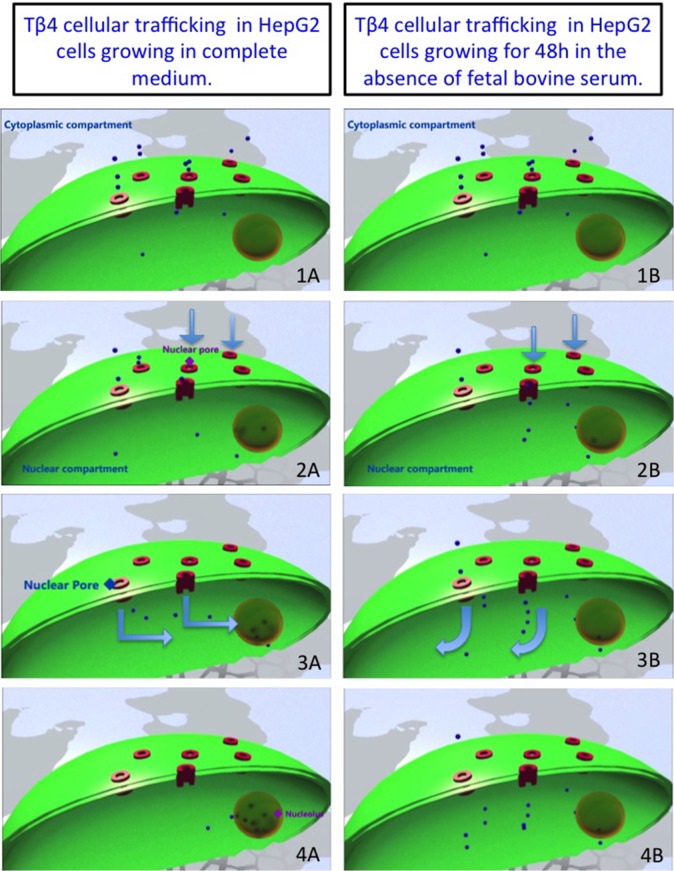
Three dimensional (3D) reconstruction of Tβ4 cellular trafficking in HepG2 cells growing at different cell conditions. 3D reconstruction of the main steps of the hypothetical Tβ4 nuclear trafficking from nucleoplasm to nucleolus in HepG2 cells growing in complete medium(1A-4A). In HepG2 cells growing for 48h in the absence of fetal bovine serum, the starvation induced stress could affect the molecular mechanisms involved in the Tβ4 cellular trafficking, preventing the localization of Tβ4 in the nucleolus (1B-4B).

In Fig. 3 we represent an hypothetical three dimensional reconstruction of Tβ4 cellular trafficking in HepG2 cells growing at different cell conditions.

Tothe best of our knowledge, this is the first time that Tβ4 presence in the nucleolus has been described, adding new fascinating physiological features to this peptide. Recently, it has been reported the presence of actin filaments in the nucleolus where they can bind to the RNA polymerase and control transcriptional processes [24]. Several actin-binding proteins have been described to be strictly associated to the nuclear actin, modulating through their actin polymerization function the RNA polymerase transcription [25]. The actin filament capping protein CapG has been suggested to control the polymerization status of the actin in the nucleolus. Due to its G-actin sequestering properties, we hypothesize that Tβ4 could contribute to regulate the length of actin filaments in the nucleolus and consequently, it could modulate nucleolar transcription processes. Although nucleolar functions are still under investigations, it has long been known that nucleolus plays a critical role in the cellular metabolism, representing a definite nuclear region where ribosome subunits are assembled and specific genes are transcripted [26]. Recently, nucleolus has been suggested to be involved in primary cell functions such as cellular stress response [23]. The different immunohistochemical Tβ4 patterns observed in the nucleoli of HepG2 cells growing in complete medium and in starving HepG2 cells could endorse this hypothesis.

A possible involvement ofTβ4 in the regulation of gene transcription was recently showed in experiments performed in glioblastoma cell linesunder starvation condition. In this study, a specificTβ4 modulation of TGFb and p53 signalling networks was related to a possible role of Tβ4 in migration, invasion, differentiation and starvation-induced cell death processes [27].

The direct involvement of Tβ4 in stress-induced processes is also suggested by the increase of mRNA expression found after 48 h of starvation (see Fig. 2).

In conclusion, our study provides new insights at ultrastructural level regarding the Tβ4 immunohistochemical pattern in the nuclei of HepG2 cells in different environmental conditions, adding new possible physiological properties to this fascinating peptide. First of all, they highlight the existence of a specific active influx from the cytoplasm into the nucleolus in HepG2 cells growing in normal conditions. Secondly, the presence of Tβ4 in the nucleoli of HepG2 cells could account for its interaction with nucleolar actin, contributing together with the other nucleolar acting binding proteins to modulate the transcription activity of the RNA polymerases. Last, but not least, Tβ4 reactivity in the nucleoplasm of starving cells could account for a specific interaction of Tβ4 with nuclear actin and its possible involvement in chromatin remodeling.
